# Muscle functional magnetic resonance imaging and acute low back pain: a pilot study to characterize lumbar muscle activity asymmetries and examine the effects of osteopathic manipulative treatment

**DOI:** 10.1186/1750-4732-3-7

**Published:** 2009-08-27

**Authors:** Brian C Clark, Stevan Walkowski, Robert R Conatser, David C Eland, John N Howell

**Affiliations:** 1 Institute for Neuromusculoskeletal Research, Ohio University College of Osteopathic Medicine, Northfield, OH, USA; 2 Department of Biomedical Sciences, Ohio University College of Osteopathic Medicine, Northfield, OH, USA; 3 Department of Family Medicine, Ohio University College of Osteopathic Medicine, Northfield, OH, USA

## Abstract

**Background:**

Muscle functional magnetic resonance imaging (mfMRI) measures transverse relaxation time (T2), and allows for determination of the spatial pattern of muscle activation. The purposes of this pilot study were to examine whether MRI-derived T2 or side-to-side differences in T2 (asymmetries) differ in low back muscles between subjects with acute low back pain (LBP) compared to asymptomatic controls, and to determine if a single osteopathic manipulative treatment (OMT) session alters these T2 properties immediately and 48-hours after treatment.

**Methods:**

Subjects with non-specific acute LBP (mean score on 1-10 visual analog score = 3.02 ± 2.81) and asymptomatic controls (n = 9/group) underwent an MRI, and subsequently the LBP subjects received OMT and then underwent another MRI. The LBP subjects reported back for an additional MRI 48-hours following their initial visit. T2 and T2 asymmetry were calculated from regions of interest for the psoas, quadratus lumborum (QL), multifidus, and iliocostalis lumborum/longissimus thoracis (IL/LT) muscles.

**Results:**

No differences were observed between the groups when T2 was averaged for the left and right side muscles. However, the QL displayed a significantly greater T2 asymmetry in LBP subjects when compared to controls (29.1 ± 4.3 vs. 15.9 ± 4.1%; p = 0.05). The psoas muscle also displayed a relatively large, albeit non-significant, mean difference (22.7 ± 6.9 vs. 9.5 ± 2.8%; p = 0.11). In the subjects with LBP, psoas T2 asymmetry was significantly reduced immediately following OMT (25.3 ± 6.9 to 6.1 ± 1.8%, p = 0.05), and the change in LBP immediately following OMT was correlated with the change in psoas T2 asymmetry (*r *= 0.75, p = 0.02).

**Conclusion:**

Collectively, this pilot work demonstrates the feasibility of mfMRI for quantification and localization of muscle abnormalities in patients with acute low back pain. Additionally, this pilot work provides insight into the mechanistic actions of OMT during acute LBP, as it suggests that it may attenuate muscle activity asymmetries of some of the intrinsic low back muscles.

## Introduction

Low back pain (LBP) is one of the most common reasons for seeking medical care, and in a given year 12-15% of the United States population will visit their physician with a complaint of back pain [1]. In recent years, between 43% and 60% of adult persons in the United States reported experiencing LBP in the previous 3-months, with the estimated annual costs for spine related conditions exceeding $200 billion [1]. The spine is an extremely complex biomechanical structure with intricate neurological, muscular, and ligamentous interfaces, and functional and structural disorders of the spine often produce symptoms affecting contiguous structures and regions. These types of disorders are frequently non-specific and referred to as neuromusculoskeletal related disorders. Conditions included in this group may include segmental disruptions, spinal sprains and strains, and other ill-defined conditions, all of which ultimately disturb and compromise the patients' functional ability and quality of life.

Numerous studies have utilized electromyography (EMG), mainly surface EMG, to examine whether patients with LBP exhibit side-to-side differences (asymmetry) in their muscle activation patterns under resting (neutral) position. The majority of these studies have not observed differences in paraspinal muscle EMG asymmetry between patients with LBP compared to controls [2-6], although some have observed differences [7]. Technical limitations of surface EMG, such as the attenuation in the myoelectric signal attributed to subcutaneous tissue and the confounds of cross talk among muscles [8,9], have limited the ability to precisely quantify and localize the muscle activity of specific lumbar muscles. Magnetic resonance imaging (MRI), on the other hand, possesses outstanding spatial resolution that allows for the investigation of individual muscles. Muscle functional MRI (mfMRI) allows noninvasive measurement of the metabolic and hemodynamic responses of skeletal muscle by observing changes in the contrast properties of certain MR images that occur in skeletal muscle with activity [10-12]. In brief, muscle activity causes an increase in skeletal muscle proton transverse relaxation times (increased T2), with T2 changes within a muscle being sensitive to as few as two repetitions of resistance exercise [13] and strongly related to the magnitude of isometric torque produced by skeletal muscle [14]. While the physiological underpinnings of these changes are complex, they primarily result from increased rates of cellular energy metabolism, which alter the image contrast properties by increasing the water content and by decreasing the intracellular pH [10]. The first purpose of this study reported here was to examine whether MRI-derived T2 or T2 asymmetries differ in skeletal muscles of the lumbar region between subjects with acute LBP compared to asymptomatic controls.

In addition to characterizing differences in paravertebral muscle activity between controls and patients with LBP, it is important to determine the effects of clinically relevant interventions on muscle activity. Manual therapies, such as spinal manipulation, mobilization and massage therapy, are used by a variety of health practitioners to treat acute LBP [15]. Osteopathic manipulative treatment (OMT) is one approach that involves the combination of numerous different manipulative techniques. A recent meta-analysis by Licciardone et al. concluded that OMT significantly reduced low back pain with the effects persisting for at least three months [16]; however, the mechanisms of action of OMT are far from understood [17]. Accordingly, a second purpose of this study was to determine if a single OMT session altered the T2 properties of the low back muscles immediately and 48-hours following the intervention.

## Methods

### Participants

Nine study participants exhibiting non-specific acute LBP and nine asymptomatic controls participated in the study. The control subjects were matched to the acute LBP subjects for age (range: 27-69 years), sex (7 women and 2 men for each group) and body mass (range: 50-86 kg) (matched within 5 years and 10 kg). Acute, rather than chronic, LBP subjects were chosen for study because it was felt that they would be more likely to respond to a single manipulative treatment than subjects with more established dysfunctions. To qualify for study inclusion the LBP subjects had to report a current acute episode of LBP that had begun within the preceding 3 weeks. When subjects inquired about the study or were referred to us we made every attempt to enroll them in the study as soon as possible. However, in many instances it required several days beyond this initial inquiry to arrange and schedule the MRI's, and on average the time from LBP onset to the first study day was 15.4 ± 2.0 days. None of the subjects reported a history of chronic LBP. Subjects were excluded if they exhibited radicular symptoms deficits (e.g., radiation of pain below the knee), if they had any bowel or bladder symptoms, had a history of spinal injuries, or had any contraindications to magnetic resonance imaging (e.g., cardiac pacemaker, metallic splinters, prostheses, etc.). Study participants were also excluded if they were less than 18 or greater than 70 years of age or weighed more than 136 kg. The LBP subjects completed an Oswestry Low Back Pain Disability Index before the experiment (mean score = 12.17 ± 3.43 (out of 50)), and rated their LBP on a 0-10 visual analog scale (mean score = 3.02 ± 2.81 cm). LBP subjects were recruited from print advertisements in the local media. The Ohio University and O'Bleness Memorial Hospital institutional review boards approved the experimental protocol, and all subjects provided written informed consent before testing.

### General Overview of the Experimental Design

**Figure 1:**
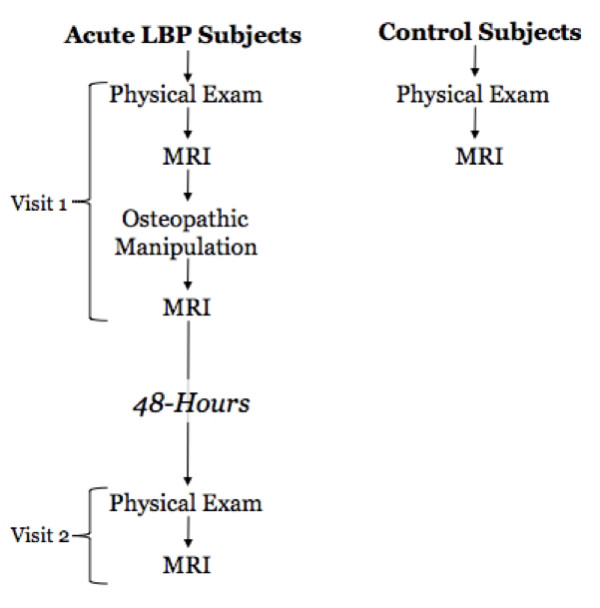
**Study timeline**.

The study design is illustrated in figure 1. In brief, the LBP subjects and the controls reported for an initial osteopathic structural examination followed by an MRI. Subsequently, the LBP subjects received OMT and then another MRI. The LBP subjects reported back to the laboratory 48-hours following their initial visit for a follow-up structural exam and MRI. One subject did not report back to the laboratory for the 48-hour post-OMT testing, and as such data for these analyses are based on 8 subjects. Detailed information of all procedures is described below.

### Physical Examination

Subjects were assessed in the standing, seated, and supine positions to evaluate for the presence of somatic dysfunction in the thoracic, lumbar, sacral, or pelvic regions. This involved a palpatory screening assessment for alterations in tissue texture change and alterations in normal regional motion, followed by more detailed palpatory diagnostic procedures designed to localize the specific dysfunctional spinal segment or segments in each of the subjects. These palpatory procedures utilized normal landmark identification in the named regions and motion testing at a segmental level to determine the extent and severity of motion restriction along with increases in tissue hypertonicity and/or tenderness to palpation.

### Muscle Functional Magnetic Resonance Imaging

**Figure 2:**
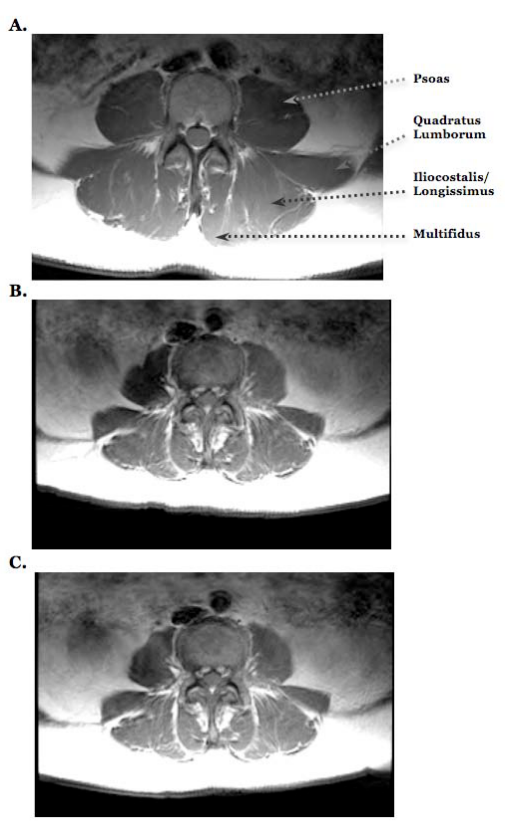
**Transaxial MRI of a 69 year old female control subject (A) and a 69 year old female with acute low back pain before (B) and immediately following osteopathic manipulation treatment (C)**. These images from the L3-L4 region illustrates the spatial orientation of the psoas, quadratus lumborum (QL), iliocostalis lumborum/longissimus thoracis, and multifidus muscles. Ten-millimeter thick transaxial images were obtained from the lumbar region with a 2000 msec repetition time, 30 and 65 msec echo times, and a 10-mm slice-to-slice interval. These derived images were used to calculate the transverse relaxation time (T2), and allows for a noninvasive measurement of the metabolic and hemodynamic responses of skeletal muscle in association with muscle activity. Note the similar signal intensity in the left and right lumbar muscles of the control subject (A), the asymmetry in the psoas and quadratus lumborum muscles before treatment (B), and the attenuation of this asymmetry immediately following treatment (C).

Standard spin-echo magnetic resonance images of the lumbar spine were obtained using a 1.5-T superconducting magnet (Genesis Sigma, GE Medical Systems, Milwaukee, WI) (Figure 2). These procedures are similar to those we have previously described [18,19]. In brief, 10-mm thick transaxial images (2000 milliseconds repetition time; 30 milliseconds and 65 milliseconds echo times; 10-mm slice-to-slice interval) were obtained from the lumbar region using a lumbar spine coil (USLS456, GE Medical Systems). During imaging, subjects were placed in the magnet bore while laying in a supine position, with legs slightly bent and resting on a foam pad allowing the lumbar spine to assume a neutral posture. Imaging procedures were identical for all of the MRI scans.

After scanning, the images were transferred to a computer for calculation of muscle T2 using the ImageJ software (Research Services Branch, National Institutes of Health). Muscle T2 was calculated on a pixel-by-pixel basis and averaged over 5-6 slices. Special care was taken to ensure the same slices were used for analysis for all of the MRI scans. The T2 values were calculated from regions of interest within the following muscles: psoas, quadratus lumborum (QL), multifidus, and the iliocostalis lumborum/longissimus thoracis (IL/LT) (these two muscles are grouped due to the difficulty in defining distinct fascial borders in some subjects). When defining regions of interest, non-muscular tissue (e.g., fat, vessels) was avoided. The dependent variables calculated from the MRI data were T2 and T2 asymmetry. T2 was calculated by averaging the left and right side values for each individual muscle, whereas T2 asymmetry is the percent difference in T2 between the sides. The coefficient of variations for the measurement error of T2 in our laboratory of manually selecting a region of interest from the respective muscles of the same image on different days in 17 subjects ranged between 1-11% (psoas 11.5 ± 1.6%, QL: 1.2 ± 0.8%, multifidus: 1.7 ± 1.4%, and IL/LT: 2.3 ± 1.6%). It should be noted that exercise-induced increases in T2 appear to persist for ~30-min [20]. The MRI was preceded by a 15-20 minute sitting rest period, along with around 10 minutes required for the structural examination. As such, the activity performed immediately before the MRI scans was standardized and minimal, and group or between-day differences of physical activity performed prior to the laboratory visit were minimized.

### Osteopathic Manipulation

Osteopathic manipulation was performed by one of two osteopathic physicians (SW or DE) who have over 15-years of clinical experience each performing osteopathic manipulation on a daily basis. Combinations of osteopathic manipulative techniques were utilized. The techniques were the ones most commonly used in the treatment of LBP, and were limited to those listed in the Glossary of Osteopathic Terminology [21]. The following treatments were commonly employed: muscle energy (assistive stretching with isometric contraction and relaxation similar to proprioceptive neuromuscular facilitation [22]), high-velocity low-amplitude thrust and articulatory release (direct joint manipulations), myofascial release (sustained pressure designed to stretch or balance tension in myofascial tissue using continuous palpatory feedback to assess tissue elasticity), and counterstrain (a technique in which the patient is placed in a position of comfort, maintained in this position for a period of time, and then is assisted in slowly returning to a neutral position). Choice of technique(s) depended upon the location and qualities of the somatic dysfunction, as well as the experience of the treating physician. The affected segments were treated until optimal improvement was obtained in reducing positional asymmetry, restriction in motion, tissue texture differences, and/or tissue sensitivity. Subjects with LBP rated their pain on a 0-10 cm visual analog scale three times: 1) before treatment (prior to the physical exam and MRI (~45 minutes before receiving OMT), 2) immediately after treatment, and 3) 48-hours after treatment. They also completed an Oswestry Low Back Pain Disability Index before treatment and 48-hours following treatment.

### Statistical Analysis

A one-way analysis of variance (ANOVA) was performed to evaluate group differences (LBP versus control) in T2 and T2 asymmetry for each muscle. In instances where we observed significant differences in T2 asymmetry LBP subjects and controls we performed additional ANOVA's to determine whether the sides with the lower and higher T2 were different between groups. A repeated measures ANOVA was performed to evaluate changes in muscle T2 asymmetry, pain, and Oswestry score over time (within-subjects factor: pre-manipulation, post-manipulation, 48-hours after manipulation). In instances where we observed significant differences in T2 asymmetry over time, additional ANOVA's were performed to determine if there were directional changes over time in the lower and higher T2 sides. Pearson's correlation coefficients were calculated to examine the relationship between T2 asymmetries and pain in the LBP group. For all analyses, a pre-set alpha level of significance equal to 0.05 was required for statistical significance, and significant main effects were followed up with a Sidak post hoc test. As stated before, this was a pilot project, and thus with its relatively small sample size rather large differences may not reach statistical significance, resulting in Type II errors. Therefore, effect sizes (here, referring to partial eta^2^, which represents the proportion of total variation attributable to the factor, partialing out other factors from the total non-error variation) are reported as an additional statistical parameter to aid in interpretation of the findings. Due to the pilot nature of this study we have denoted instances where significance was not reached but modest effect sizes were observed (eta^2 ^≥ 0.15). The SPSS statistical package (version 17.0 for Mac, Chicago, IL) was used for data analysis. Data are presented as means+SEM, unless otherwise stated.

## Results

### T2 Differences Between Acute Low Back Pain Subjects versus Controls

**Figure 3:**
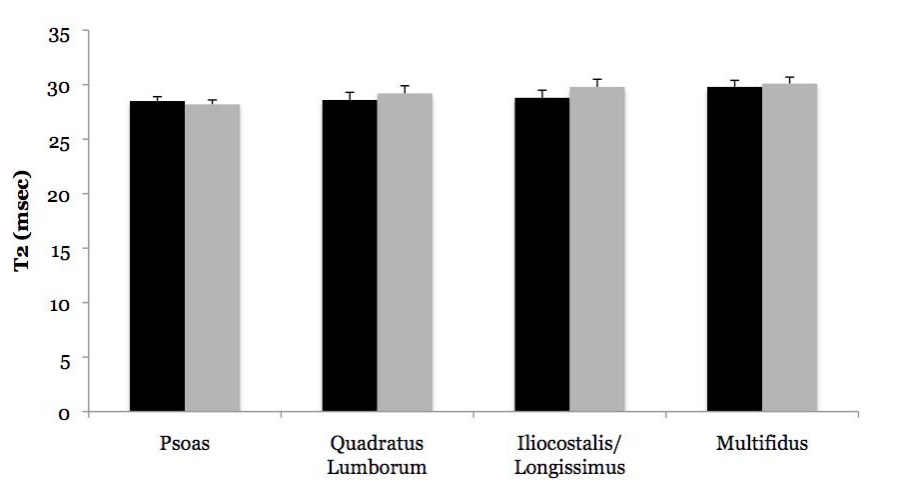
**Comparison of absolute T2 averaged from the left and right side muscles between subjects with acute low back pain (LBP; black bars) and asymptomatic controls (grey bars)**. No differences were observed between LBP subjects and controls.

**Figure 4:**
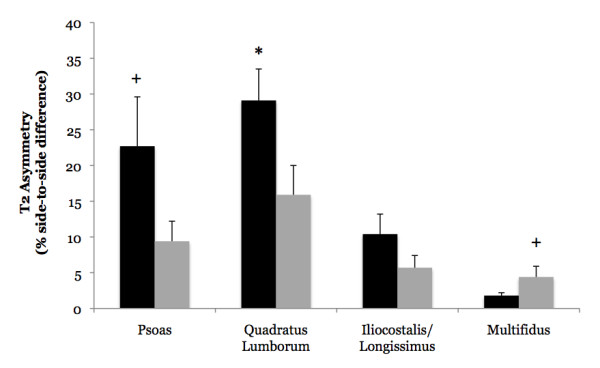
**Comparison of T2 asymmetry between subjects with acute low back pain (LBP; black bars) and asymptomatic controls (grey bars)**. T2 asymmetry was calculated as the absolute value of the percent difference in MRI-derived T2 between the left and right side muscles. The quadratus lumborum displayed a significantly greater T2 asymmetry in LBP subjects when compared to controls (*p = 0.05, eta^2 ^= 0.23). Similarly, the psoas muscle exhibited a modest effect size for greater T2 asymmetry in LBP subjects when compared to controls (+ eta^2 ^= 0.16), although this difference failed to reach significance (p = 0.11). The multifidus muscle also exhibited a modest effect size for between-group differences with the LBP subjects exhibiting a lesser degree of T2 asymmetry in relation to controls (+ eta^2 ^= 0.17), although this difference failed to reach significance (p = 0.11).

No differences were observed between LBP subjects and controls when T2 was averaged for the left and right side muscles (Figure 3) (Psoas: 28.5 ± 0.4 vs. 28.2 ± 0.4 msec, p = 0.58, eta^2 ^= 0.02; QL: 28.6 ± 0.7 vs. 29.2 ± 0.7 msec, p = 0.54, eta^2 ^= 0.03; IL/LT: 28.8 ± 0.7 vs. 29.8 ± 0.7 msec, p = 0.34, eta^2 ^= 0.06; Multifidus: 29.8 ± 0.6 vs. 30.1 ± 0.6, p = 0.74, eta^2 ^= 0.01). Differences in T2 asymmetry were observed, however, between LBP subjects and controls (Figure 4). Specifically, the QL displayed a significantly greater T2 asymmetry in LBP subjects when compared to controls (29.1 ± 4.3 vs. 15.9 ± 4.1%; p = 0.05, eta^2 ^= 0.23). All of the LBP subjects displayed QL asymmetry values greater than that of the average control subject (> 15%), and in 8 of these 9 subjects the right side exhibited the higher T2 value. Further analysis indicated that, compared to controls, the LBP subjects exhibited a reduced T2 on the side with the lower T2 value (25.0 ± 0.5 vs. 27.1 ± 0.7 msec; p = 0.03, eta^2 ^= 0.27), but that no difference existed for the side with the higher T2 value (32.3 ± 0.9 vs. 31.3 ± 1.1 msec; p = 0.52, eta^2 ^= 0.03). The psoas muscle exhibited a modest effect size for greater T2 asymmetry in LBP subjects when compared to controls (22.7 ± 6.9 vs. 9.5 ± 2.8%; eta^2 ^= 0.16), although this difference failed to reach significance (p = 0.11). Six of the LBP subjects displayed psoas asymmetry values greater than that of the average control subject (> 9%), and in all of these LBP patients the higher T2 value was associated with the right side muscle. The multifidus muscle also exhibited a modest effect size for between-group differences with the LBP subjects exhibiting a lesser degree of T2 asymmetry in relation to controls (1.8 ± 0.4 vs. 4.4 ± 1.5%; eta^2 ^= 0.17), although this difference failed to reach significance (p = 0.11). The T2 asymmetry for the IL/LT was similar between LBP subjects and controls (10.4 ± 2.8 vs. 5.7 ± 1.7%; p = 0.19, eta^2 ^= 0.11).

### The Effect of OMT on T2 Asymmetry in Acute LBP Subjects

**Figure 5:**
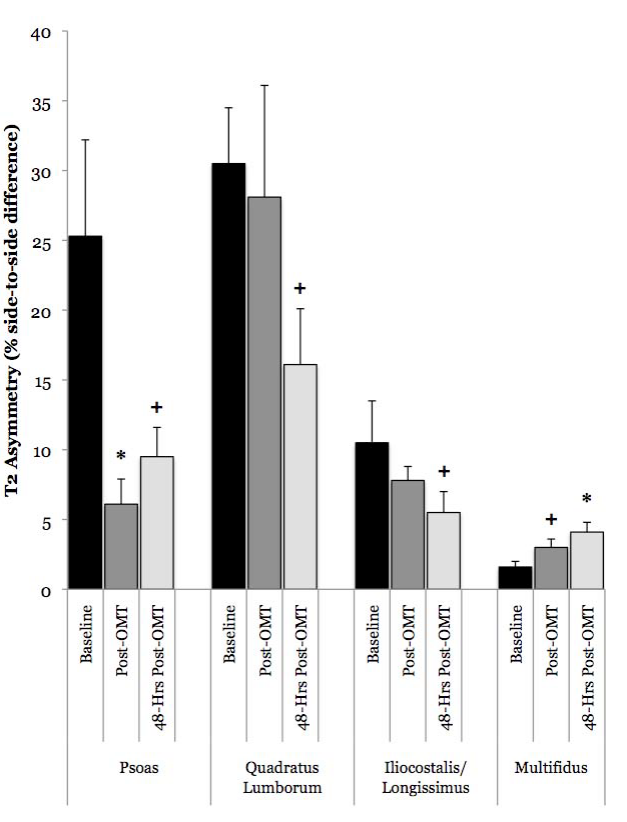
**Changes in T2 asymmetry in subjects with acute low back pain (LBP) following a single osteopathic manipulative treatment (OMT) session**. Data were obtained before manipulation (baseline), immediately following OMT and 48-hours following OMT. T2 asymmetry was calculated as the absolute value of the percent difference in MRI-derived T2 between the left and right side muscles. Immediately following OMT the T2 asymmetry in the psoas muscle was reduced, but it returned to baseline levels after 48-hours although a modest effect size was still observed (+p = 0.06, eta^2 ^= 0.41). Conversely, 48-hours following OMT a small, but significant, increase in multifidus T2 asymmetry was observed (*p = 0.01, eta^2 ^= 0.67). The quadratus lumborum and iliocostalis/longissimus muscles exhibited modest effect sizes for reduced T2 asymmetry associated with OMT (+ eta^2 ^= 0.19 and 0.17, respectively), although these differences failed to reach significance (p = 0.23 and 0.27, respectively).

**Figure 6:**
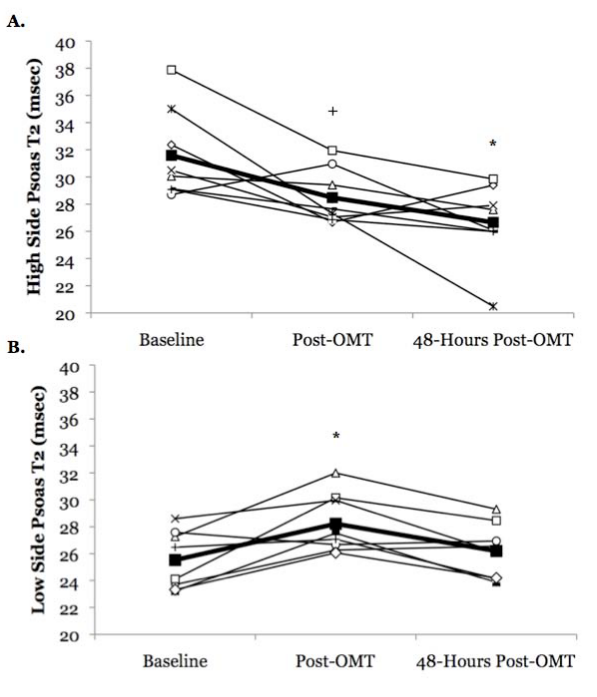
**Changes in psoas muscle T2 for the side with a higher T2 at baseline (A) and the side with a lower T2 at baseline (B) in subjects with acute low back pain following a single osteopathic manipulative treatment (OMT) session**. Data are plotted for individual subjects (open symbols) along with the mean response (filled square symbols). Data were obtained before manipulation (baseline), immediately following OMT and 48-hours following OMT. We observed a significant change in psoas T2 asymmetry following OMT. Follow-up analysis indicated that the side with the higher initial T2 value did not change immediately following OMT despite exhibiting a modest effect size (+eta^2 ^= 0.51), but that it was reduced 48-hours after OMT (*p = 0.04, eta^2 ^= 0.61). The side with the lower initial T2 value increased immediately following OMT (*p = 0.04, eta^2 ^= 0.61), and returned back to baseline levels after 48-hours.

OMT resulted in a significant reduction in psoas T2 asymmetry (treatment main effect p = 0.03, eta^2 ^= 0.39) (Figure 5). Psoas T2 asymmetry was significantly reduced when compared to baseline immediately following OMT (25.3 ± 6.9 to 6.1 ± 1.8%, p = 0.05, eta^2 ^= 0.42), but it returned to baseline levels after 48-hours although a modest effect size was still observed (9.5 ± 2.1%, p = 0.06, eta^2 ^= 0.41). Further analysis indicated that the OMT resulted in an increase in T2 on the side with the lower initial T2 value immediately following OMT (25.5 ± 0.5 to 28.2 ± 0.8 msec; p = 0.04, eta^2 ^= 0.61) that returned back to baseline levels after 48-hours (26.2 ± 0.7 msec; p = 0.84, eta^2 ^= 0.08). The side with the higher initial T2 value did not decrease significantly immediately following OMT (31.1 ± 1.2 to 28.5 ± 0.7 msec; p = 0.09) although a modest effect size was observed (eta^2 ^= 0.51); however, 48-hours post-OMT this side was significantly reduced (26.7 ± 1.0 msec; p = 0.04, eta^2 ^= 0.61) (Figure 6). Conversely, OMT resulted in a significant increase in multifidus T2 asymmetry (treatment main effect p = 0.04, eta^2 ^= 0.36) (Figure 5). Multifidus T2 asymmetry was not affected immediately following manipulation (1.6 ± 0.4 to 3.0 ± 0.6%, p = 0.15, eta^2 ^= 0.19), but it was slightly increased 48-hours later (4.1 ± 0.7%, p = 0.01, eta^2 ^= 0.67). Further analysis did not yield any information on the direction of effect, as neither the lower side nor the higher side exhibited significant changes following OMT (Lower Side- Baseline: 29.4 ± 0.6 msec, Immediately post-OMT: 29.5 ± 0.7 msec, 48-hours post-OMT: 29.9 ± 0.9 msec; p = 0.66, eta^2 ^= 0.06; Higher Side- Baseline: 29.9 ± 0.7 msec, Immediately post-OMT: 29.6 ± 1.0 msec, 48-hours post-OMT: 29.6 ± 0.7 msec; p = 0.84, eta^2 ^= 0.02). The QL and IL/LT muscles did not display significant changes in T2 asymmetry with OMT (QL treatment main effect p = 0.23; IL/LT treatment main effect p = 0.27), although modest effect sizes for a reduction were observed (QL: Baseline: 30.5 ± 4.4, Immediately post-OMT: 28.1 ± 7.8, 48-Hrs Post: 16.1 ± 4.2, eta^2 ^= 0.19; IL/LT: Baseline: 10.5 ± 3.0, Immediately post-OMT: 7.8 ± 1.1, 48-Hrs Post: 5.5 ± 1.5, eta^2 ^= 0.17) (Figure 5).

With regards to patient perceptions of the effects of treatment, OMT resulted in a significant reduction in self-reported pain (treatment main effect p = 0.01, eta^2 ^= 0.61). Pain was significantly reduced immediately following OMT (3.36 ± 0.99 to 1.68 ± 0.56 cm, p = 0.01, eta^2 ^= 0.62), and it remained reduced 48-hours later (1.90 ± 0.63 cm, p = 0.01, eta^2 ^= 0.61). The Oswestry Low Back Pain Disability Index score was also reduced 48-hours after treatment (12.17 ± 1.14 to 3.93 ± 1.31; p = 0.01, eta^2 ^= 0.77).

### Relationship between LBP and T2 Asymmetry

We observed a significant correlation between pain (as assessed via the visual analog scale) and psoas T2 asymmetry at baseline (before OMT) (*r *= 0.76, p = 0.02). Additionally, we observed that the change in pain immediately and 48-hours following OMT was correlated with the change in T2 asymmetry (immediately post-OMT: *r *= 0.75, p = 0.02; 48-hours post-OMT: *r *= 0.85, p = 0.01). No other significant correlations were observed between pain and T2 asymmetry in other muscles (p > 0.05). As stated above, for the psoas muscle six of the LBP subjects displayed asymmetry values greater than that of the average control subject (> 9%), and in all of these LBP patients the higher T2 value was associated with the right side muscle. Interestingly, in all six of these subjects the L4 or L5 vertebrae were rotated left. We did not observe any association between the side with the higher T2 asymmetry and self reported localization of LBP, as 3 subjects reported pain on the left side, 1 reported pain on both sides, 3 reported centralized pain, and 2 reported pain on the right side.

## Discussion

The purpose of this work was to determine if MRI-derived T2 asymmetries in the low back muscles, an index of side-to-side variation in muscle activation, differed between acute LBP subjects and asymptomatic controls. Magnetic resonance imaging possesses outstanding spatial resolution; thus, we were able to separate the individual muscles of the lumbar spine. As this was an exploratory pilot project, strong conclusions cannot be made; however, our findings do suggest that our LBP subjects exhibited greater asymmetry in the muscle activation level of the QL and possibly of the psoas muscle. A second aim of this study was to determine if an intervention involving OMT altered T2 asymmetries. The most striking observation was that the OMT dramatically reduced the muscle activity asymmetry of the psoas muscle, and that this reduced asymmetry is associated with reduced LBP. This pilot work demonstrates the feasibility of using muscle functional MRI for quantification and localization of muscle abnormalities in individuals with acute low back pain. The precise quantification of specific muscle abnormalities associated with LBP will allow for better evaluation of interventional treatments (e.g., manual therapies, pharmacologic therapies, etc).

Recently, the use of mfMRI has begun to extend beyond the scope of skeletal muscle and exercise physiology, and has been applied as a tool to examine pathologic muscle. For example, we have used it to examine muscle activity in spastic elbow flexor muscles following stroke [19], and others have used it to examine muscular properties of the lateral pterygoid in patients with TMJ [23]. Thus, based on exercise studies indicating that changes in T2 are primarily due to an increased rate of cellular energy metabolism [10-12], our observation of greater T2 asymmetries in the QL of LBP subjects suggests that these individuals have differences in their degree of unilateral muscle activity or metabolic state. Our observations also suggest that the psoas muscle, based on our observed effect size, may also exhibit greater asymmetrical muscle activity in LBP subjects. Although LBP subjects had a tendency to have lower degrees of muscle activity asymmetry in the multifidus, the magnitude of difference was so nominal (2% vs. 4%) that it is difficult to imagine that it holds substantial clinical significance.

Our observation of asymmetrical activation patterns of the lumbar paraspinal muscles in patients with LBP is contrary to numerous studies approaching this research question using EMG [2-6]. In contrast to the present study, all of these studies focused on chronic LBP subjects, excluding the type of subjects used in this study. At least one study (7), which analyzed the overall pattern of back muscle EMG with multi-electrode arrays, did report asymmetry in LBP subjects. It is likely that technical limitations of conventional surface EMG, such as the inability to provide an indication of specific muscle patterns [8], variability in the myoelectric signal attributed to subcutaneous adipose tissue [24], and electrode type and placement [25], along with confounding of the myoelectric signal by cross talk among muscles [26], have made it difficult to make definitive conclusions about lumbar muscle activity. Thus, it seems likely that our ability to detect muscle activity asymmetries is largely due to magnetic resonance imaging possessing outstanding spatial resolution that allowed us to investigate individual muscles, including deep muscles like the psoas, from which EMG signals are difficult to obtain unequivocally.

We also observed that OMT decreased T2 asymmetry with the most notable reduction occurring in the psoas muscle. It has long been postulated that the mechanism(s) of OMT are related to an attenuation of the gain of the muscle spindle afferents that reduces reflexive contractile activity [27,28]. Indeed we did observe that in the side exhibiting the greater baseline T2 that OMT resulted in a reduction in T2 activity. Additionally, it also appeared to increase T2 on the side with the lower baseline T2. Thus, while these data do not provide insight into specific neurologic mechanisms of OMT they do suggest that in acute LBP OMT may function to normalize psoas muscle activity by reducing the activity in the hyperactive side and increasing the activity in the hypoactive side. Interestingly, Ellestad and colleagues observed OMT reduced the amplitude of the interference EMG signal during a lumbar extension task in both controls and patients with LBP [29], and Lehman and McGill reported that spinal manipulation decreased EMG amplitude of both paraspinal and abdominal muscles [30]. Thus, when these findings are collectively considered it seems that one plausible hypothesis for the mechanistic action of manipulative therapies is that they reduce the activity of hyperactive muscles with a net result being the normalization of muscle paraspinal muscle activity and side-to-side balance. However, it is not possible to know if muscle activity asymmetries are primary causes of acute LBP; it is possible that our observed responses are secondary to injury or other musculoskeletal problems.

It could be argued that other characteristics of skeletal muscle, beyond the activity state of the muscle, could explain our T2-related findings. For example, the lumbar muscles of chronic LBP subjects exhibit muscle atrophy [31], and muscle atrophy is associated with a concomitant increase in inter- and intra-muscular adipose tissue which would affect T2 values [32,33]. Although the data are not included, muscle cross sectional areas were measured in this study, and no differences were detected either between LBP subjects and controls or within LBP subject before and after treatment. Muscle pathologies such as nodular fasciitis or myositis ossificans as well as differences in muscle fiber types [36] could also alter T2 signal intensities [34,35],. These explanations seem highly unlikely, however, as: i) we avoided non-muscular tissue in selecting our regions of interest, ii) all MR scans were examined by a radiologist for pathologic findings, and iii) the T2 asymmetry in the psoas muscle was immediately reduced following the manipulative intervention. The observation of an immediate reduction in T2 asymmetry following OMT suggests that the physiologic explanation of T2 was most probably related to the activity (metabolic state) of the muscle, as pathologic findings and/or fiber types would not change in the short-term [37]. Accordingly, it seems that one explanation of the QL T2 asymmetry difference between LBP subjects and controls relates to the LBP subjects having differences in unilateral muscle activity, with one side appearing to be hypoactive; the mean T2 value on the side with the lower T2 was only 25 msec. One potential explanation of hypoactivity could be arthrogenic muscle inhibition, as joint damage has been associated with greater inhibition [38].

The findings from the present study raise several other pertinent questions as to the relative contribution of individual muscle activity asymmetries to the etiology of non-specific LBP and to the efficacy and mechanism of OMT in treating acute LBP. For example, we observed a modest effect size for differences in psoas T2 asymmetry and controls, a significant correlation between LBP and psoas T2 asymmetry, and that the subjects with high absolute T2 values on the right side exhibited L4 or L5 vertebrae that were rotated left. The latter finding is particularly interesting when one considers the functional anatomy and structural diagnosis of the lumbar spine. The segmental diagnosis of left rotation implies a relative tendency of a vertebral body to prefer rotation to the left and restriction of rotation to the right. The palpatory and motion testing reveals a relatively posterior transverse process on the left, suggesting an anterior transverse process on the right (counterclockwise rotation of the vertebral body when viewed from above). Thus, it is possible that the right anterior displacement is due to the hyperactive musculotendinous unit pulling on the pulling on the transverse process.

To fully address these questions, we must consider them within the context of the present study's limitations. With regards to the role of individual muscle asymmetry in contributing to LBP, we did observe that the QL exhibited greater T2 asymmetry than asymptomatic controls, and that the psoas muscle displayed a relatively large, albeit non-significant, mean difference. We must caution, however, against interpreting these findings as an indication that muscle activity asymmetry patterns in these given muscles causes or contributes to acute LBP as the observation of a difference between groups does not indicate causality. Thus, further work is needed to fully elucidate the extent and impact of muscle asymmetry patterns of the individual components of the lumbar muscles in individuals with acute LBP. In the present work our study population consisted mostly of subjects with mild, acute LBP (e.g., mean Oswestry score of ~12 out of 50 and pain scores of ~3 out of 10), and it is suggested that subsequent work investigate muscle asymmetries in a cohort with more severe, acute LBP. Additionally, we should also reiterate that our study examined subjects with acute LBP, and that these findings should not be extrapolated to individuals with chronic LBP.

We must also express caution when interpreting our data on the efficacy and mechanisms of OMT. While we did observe that LBP subjects reported an immediate reduction in pain with persistent effects two days following treatment and that the reduction in pain was associated with reduced T2 asymmetry, it should be noted that we did not have appropriate comparison groups to fully allow for the assessment of clinical efficacy or mechanisms of action (e.g., natural time course, active treatment and/or sham groups). Additionally, the present study utilized a combination of OMT techniques, making it impossible to know which individual techniques had the primary effects, or if, indeed, a combination of techniques is necessary for the observed effects.

## Conclusion

This pilot study utilized muscle functional magnetic resonance imaging to examine if T2 asymmetries in the low back muscles, an index of side-to-side variation in muscle activation, differed between subjects with acute LBP and controls, and to determine if OMT altered T2 asymmetries. We observed that subjects with acute LBP had a greater T2 asymmetry in the quadratus lumborum. In the subjects with LBP, OMT resulted in an immediate reduction of T2 asymmetry in the psoas muscle, and this reduction in T2 asymmetry was associated with reduction in pain. Collectively, this pilot work shows the feasibility of employing muscle functional MRI for quantification and localization of muscle abnormalities in patients with acute low back pain. The implications for clinical research could be immense as the precise quantification of specific muscle abnormalities associated with LBP would allow for better evaluation of interventional treatments (e.g., manual therapies, pharmacologic therapies, etc). Additionally, this pilot work provides insight into the mechanism(s) of action of OMT during acute LBP, as it suggests that OMT may function to attenuate muscle activity asymmetries of some of the intrinsic low back muscles.

## Competing interests

The authors declare that they have no competing interests.

## Authors' contributions

BC was responsible for overseeing the development and implementation of the MRI aspects of the study and drafted the manuscript. SW performed around half of the OMT sessions and structural examinations. RC coordinated subject recruitment and scheduling and performed the data analyses. DE performed around half of the OMT sessions and structural examinations. JH conceived of the study, and directed its overall design and coordination. All authors read and approved the final manuscript.
